# Contemporary Percutaneous Coronary Intervention in Diabetic Patients

**DOI:** 10.31083/RCM44861

**Published:** 2025-12-22

**Authors:** Francesco Tartaglia, Gaia Filiberti, Valentina Bernardini, Mauro Gitto, Pier Pasquale Leone, Azeem Latib, Damiano Regazzoli, Giulio Stefanini, Antonio Mangieri, Antonio Colombo

**Affiliations:** ^1^Department of Biomedical Sciences, Humanitas University, 20072 Pieve Emanuele, Milan, Italy; ^2^IRCCS Humanitas Research Hospital, Cardio Center, 20089 Rozzano, Milan, Italy; ^3^Division of Cardiology, Montefiore Medical Center, Bronx, NY 10467, USA

**Keywords:** diabetes, revascularization, percutaneous coronary intervention, drug-eluting stent, drug-coated balloon

## Abstract

Coronary artery disease is a leading cause of morbidity and mortality in patients with type 2 diabetes mellitus. Indeed, diabetic patients often present with silent or atypical symptoms and are more likely to develop complex, diffuse, rapidly progressive, and recurrent atherosclerosis. While current guidelines favor coronary artery bypass grafting in diabetic patients with multivessel disease, advances in percutaneous coronary intervention technology have broadened the range of revascularization options for this high-risk population. Nevertheless, despite major improvements in stent platforms over the past two decades, diabetic patients continue to experience higher rates of in-stent restenosis and adverse cardiovascular events compared to non-diabetics, in part, because of the permanent metallic scaffold. Therefore, novel strategies, including drug-coated balloons, minimize chronic inflammation and eliminate permanent vessel caging, thereby offering promising alternatives in this setting, particularly for lesion subsets typical of diabetic patients. This review discusses the current landscape and future directions of percutaneous coronary revascularization in diabetic patients, outlining the evolution from drug-eluting stents to emerging metal-sparing technologies, and highlighting the persistent challenges in achieving optimal outcomes in this population.

## 1. Introduction

Coronary artery disease (CAD) is the leading cause of morbidity and mortality in patients with type 2 diabetes mellitus (T2DM) [1]. Compared to non-diabetic individuals, patients with T2DM face a two- to fourfold increased risk of developing CAD, which is typically anatomically complex and rapidly progressive [2].

Current European Society of Cardiology (ESC) guidelines recommend coronary artery bypass grafting (CABG) for diabetic patients with multivessel disease (MVD) (Class IA), particularly when the disease involves the left main or proximal left anterior descending artery (LAD) [3, 4]. Nevertheless, technological advancements in percutaneous coronary intervention (PCI) have significantly improved procedural success and long-term outcomes, and PCI has become a viable alternative in selected diabetic patients. PCI is now recommended for high-risk patients who are not candidates for CABG and is acceptable as first-line therapy in those with less extensive disease and low anatomical complexity [4].

In this review, we aim to explore the interventional treatment of CAD in diabetic patients, with a focus on new technologies for PCI.

## 2. Features of CAD in Diabetic Patients

Diabetes accelerates atherogenesis through several mechanisms [5]: chronic inflammation leads to increased oxidative stress, while persistent hyperglycemia promotes non-enzymatic glycation of proteins, causing endothelial dysfunction and a prothrombotic state [6]. Additionally, elevated endothelin levels induce vasoconstriction, and increased matrix metalloproteinase activity destabilizes atherosclerotic plaques, resulting in an increased risk of acute coronary syndromes (ACS) [7]. Moreover, diabetic dyslipidemia—characterized by elevated triglycerides, reduced high-density lipoprotein cholesterol, and increased low-density lipoproteins—promotes the development of diffuse disease and impairs collateral vessel formation [8].

### 2.1 Plaque Morphology and Clinical Implications

Coronary plaques in diabetic patients more frequently present high-risk features, including large necrotic cores, high inflammatory cell infiltration, and advanced calcification, than in non-diabetic individuals [9]. These histopathological findings have been confirmed by studies using optical coherence tomography (OCT) or intravascular ultrasound (IVUS), reporting a high frequency of plaques with thin-cap fibroatheromas (TCFA), which are associated with an increased risk of adverse events [10] and positive remodeling [11]. Recently, preventive PCI of high-risk vulnerable plaques in diabetic patients showed reduced revascularizations and hospitalizations compared with optimal medical therapy, but had no impact on target vessel myocardial infarction or cardiac mortality, suggesting that systemic factors may outweigh focal plaque features in determining residual risk [12].

### 2.2 Angiographic Patterns of CAD in Diabetic Patients

**Fig. 1.**
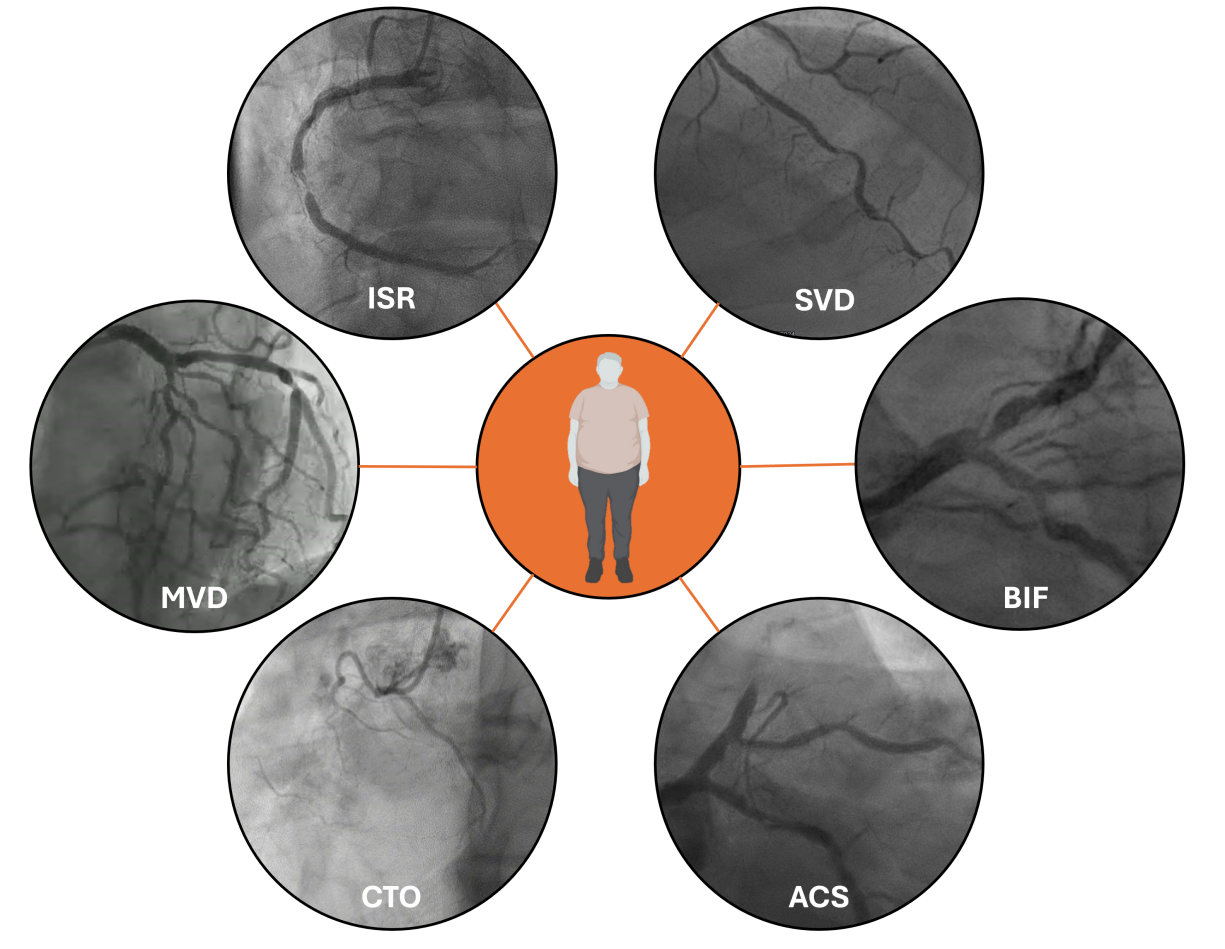
**Typical patterns of coronary artery disease in diabetic patients**. Legend: ISR, in-stent restenosis; SVD, small vessel disease; BIF, Bifurcation lesion; ACS, acute coronary syndromes; CTO, Chronic Total Occlusion; MVD, multivessel disease.

CAD in patients with DM presents distinct angiographic features (Fig. 1). T2DM increases total plaque burden, leading to a more frequent development of both critical and subcritical stenoses, which may serve as vulnerable sites for future MI. Consequently, MVD is found in 40–80% of patients with DM and CAD [13, 14].

Vessels of patients with DM also undergo structural changes. Coronary arteries have small diameters, partly due to reduced nitric oxide–mediated vasodilation as well as impaired angiogenesis [15]. The diffuse extent of atherosclerosis leads to a higher incidence of small vessel disease (SVD) [13, 14] and bifurcation involvement, frequently characterized by extensive side-branch involvement, resulting in a high SYNTAX (SYNergy between PCI with TAXUS and Cardiac Surgery) score.

Aggressive disease also determines a high rate of in-stent restenosis (ISR) after stenting, with aggressive neointimal hyperplasia and neoatherosclerosis that tends to localize on stent edges [16, 17].

### 2.3 From Silent Ischemia to ACS: The Multiple Faces of CAD in Diabetes

In contrast to non-diabetic individuals, only a fraction of diabetic patients with CAD experience typical angina [14], whereas the absence of symptoms or presence of atypical ones (i.e., dyspnea, exertional fatigue, nausea, and diaphoresis) is common due to autonomic neuropathy, which blunts pain perception [18]. Hence, medical attention is frequently delayed, and silent myocardial infarctions are frequent [19], leading to a worse long-term prognosis [20]. On the other hand, diabetes poses an increased risk of ACS: one out of four patients with ST-elevation myocardial infarction (STEMI) has a history of T2DM, and almost half are newly diagnosed with diabetes or prediabetes, which confers a significantly higher risk of short-term adverse events [21].

## 3. Revascularization of CAD in Diabetic Patients: Indications and Technical Issues

### 3.1 Choosing the Optimal Revascularization Strategy: CABG Versus PCI

Despite major advancements in PCI techniques and refinements in stent platforms, patients with DM continue to experience poorer clinical outcomes than the general population [22]. The enhanced risk of ISR and repeat target lesion revascularization (TLR) [23, 24, 25] is one of the main reasons current guidelines recommend CABG over PCI in patients with DM and chronic MVD [4, 26], especially in young patients. In the FREEDOM trial, CABG was associated with significantly lower rates of death and MI compared to PCI at the 8-year follow-up [13]. Similarly, the SYNTAX trial showed higher 5-year all-cause mortality with PCI compared to CABG (*p* = 0.02), although this difference was no longer significant at 10 years (*p* = 0.29) [27]. Whether the improved outcomes associated with contemporary PCI—driven by advanced tools and the routine use of intravascular imaging and physiology guidance—will ultimately narrow the gap with surgery in patients with T2DM remains uncertain. In the SYNTAX II study, physiology-guided PCI showed comparable outcomes to the CABG cohort of the SYNTAX trial [28]; however, this result should be confirmed in properly designed randomized controlled trials (RCTs).

In the meantime, PCI with drug-eluting stents (DES) remains an acceptable alternative for patients with less extensive disease (i.e., single-vessel disease or two-vessel disease not involving the left anterior descending, and those with a SYNTAX score ≤22). The role of the SYNTAX score in guiding revascularization strategy in patients with DM and MVD remains a topic of debate [29]. Because it relies exclusively on angiographic assessment of lesion complexity, the SYNTAX score fails to capture the biological complexity of diabetic atherosclerosis and provides no information on plaque vulnerability. To date, however, no alternative validated scoring systems are available to guide the choice between CABG and PCI. As such, the SYNTAX score remains the reference tool for revascularization decisions.

### 3.2 Role of Intravascular Imaging and Functional Assessment in Diabetic PCI

Although recent evidence suggests diabetic status does not affect the benefit of intravascular imaging (IVI), the prognostic impact of routine IVI guidance in patients with DM is currently being addressed in a dedicated RCT (NCT06380868). Similarly, fractional-flow reserve (FFR) should be used regardless of the presence of diabetes: the recent results of the FAME (Fractional Flow Reserve vs Angiography for Multivessel Evaluation) 3 trial showed that, in patients with T2DM and MVD, outcomes were similar between PCI and CABG in those with low SYNTAX scores (<23), whereas higher scores (≥23) were consistently associated with worse outcomes following PCI [30]. FFR and instantaneous wave-free ratio (iFR) led to comparable results in patients with DM [31].

Although physiology and IVI have had different roles (the former to determine the need for PCI, and the latter to guide it), they were compared in an RCT that found similar outcomes at 2 years in both diabetic and nondiabetic patients [32]. More appropriately, a combination of the two methodologies has been tested in the COMBINE OCT-FFR (Optical Coherence Tomography Morphologic and Fractional Flow Reserve Assessment in Diabetes Mellitus Patients) trial, which reported that 44% of patients with DM, intermediate lesions, and FFR >0.80 had TCFAs, and that their presence was associated with a 4.7-fold increased risk of major adverse cardiovascular events (MACE) [10]. These findings suggest that plaque characterization with OCT may help refine risk stratification and decision-making in patients with DM undergoing PCI, even beyond physiologic measurements. However, no definitive recommendation favoring interventional treatment over medical therapy can be drawn from this evidence. The ongoing COMBINE-INTERVENE (COMBINED Ischemia and Vulnerable Plaque Percutaneous Intervention to Reduce Cardiovascular Events, NCT05333068) trial will address this knowledge gap by comparing interventions based on functional ischemia only versus functional ischemia plus OCT assessment.

### 3.3 Antiplatelet Therapy: Duration and Special Considerations

Notably, T2DM is considered a thrombotic risk factor, but patients with DM frequently have associated comorbidities (e.g., renal dysfunction and polypharmacy), which make them a high bleeding risk population, necessitating careful consideration of dual antiplatelet therapy (DAPT) composition and duration. Recent evidence suggests that a short DAPT (1–3 months) followed by P2Y_12_ inhibitor monotherapy may reduce bleeding without increasing ischemic complications in this population compared to standard DAPT [33]. This finding was confirmed by two recent large-scale meta-analyses, in which diabetic status did not diminish the benefit of this alternative antiplatelet regimen [34, 35]. Further insights on P2Y_12_ monotherapy are underway (NCT04484259).

## 4. Drug-Eluting Stents

### 4.1 Impact of Stent Technology on Outcomes in Diabetic Patients

DES have replaced bare-metal stents (BMS) and plain old balloon angioplasty (POBA) because of their superior efficacy in limiting neointimal proliferation and reducing the need for TLR [32, 36, 37]. From a technical standpoint, DES consists of a metallic scaffold, an antiproliferative drug (paclitaxel or a -limus derivative), and a drug carrier matrix—usually a polymer coating—that controls drug release.

New-generation DES—with thinner struts and improved deliverability—have improved both safety and efficacy in the general population by reducing local inflammation and thrombogenicity [38, 39, 40]. Nevertheless, technological progress has led to only modest clinical benefit in diabetic patients. In fact, T2DM negatively impacts DES performance, with an annual rate of adverse events almost doubled in patients with DM as compared to nondiabetic ones [41]. A recent intravascular imaging study found a lower minimum neointimal coverage grade and a higher prevalence of uncovered stent struts in diabetic versus nondiabetic patients during early follow-up after DES implantation [42]. Hence, T2DM and insulin dependence remain strong predictors of MACE after PCI [23, 24, 43, 44].

Table 1 (Ref. [23, 32, 45, 46, 47, 48, 49, 50, 51, 52, 53, 54, 55, 56, 57, 58, 59, 60, 61, 62, 63, 64, 65, 66, 67, 68, 69, 70, 71, 72, 73, 74, 75, 76, 77, 78, 79, 80, 81]) summarizes clinical outcomes associated with different DES technologies in patients with DM.

**Table 1.** **Main randomized and observational evidence on DES in DM**.

Study	Study type	Year	Setting	Treatment	Control	Patients in the treatment arm (N) with % of DM and IDDM	Patients in the control arm (N) with % of DM and IDDM	Follow-up, months	Outcomes in diabetic patients (unless further specified)
FIRST GENERATION DES									
Paclitaxel-eluting stents (PES)									
TAXUS IV [45]	RCT	2005	Single *de novo* lesion in native CAD	PES	BMS	662	652	9	∙ TVR: 11.3% (PES) vs 24% (BMS) (HR: 044, 95% CI 0.25–0.78, *p* = 0.004)
						DM: 23.4%	DM: 25%		∙ TVF: 15% vs 27.2 % (HR: 0.52, 95% CI 0.31–0.86, *p* = 0.0095)
						IDDM: 7.7%	IDDM: 8.3%		∙ MACE: 15.6% vs 27.7% (HR: 0.53, 95% CI 0.32–0.87, *p* = 0.01)
									∙ IDDM vs non-IDDM vs nondiabetics: 13.4% vs 19.6% vs 26.2%, *p* = 0.0003
									∙ Restenosis: IDDM vs non-IDDM: 42.9% vs 29.7% vs 24.4%, *p* = 0.17
TAXUS Clinical Program [60]	Pooled analysis of TAXUS I, II, IV, V trials	2008	Single *de novo* lesion in native CAD	PES	BMS	1755	1758	48	∙ TLR: 12.4% (PES) vs 24.7% (BMS), *p* = 0.0001
						DM: 23.2%	DM: 23.8%		∙ TVR: 24.4% vs 30.2%, *p* = 0.005
						IDDM: 7.2%	IDDM: 7.8%		∙ ST: 1.4% vs 1.2%, *p* = 0.92
									∙ MI: 6.9% vs 8.9%, *p* = 0.17
									∙ Death: 8.4% vs 10.3, *p* = 0.61
									∙ Comparable results between IDDM vs non-IDDM
TAXUS ATLAS Program [61]	Pooled analysis of TAXUS ATLAS (Taxus Atlas, Small Vessel, Long Lesion, Direct Stent)	2009	*De novo* lesions	PES	NA	1529	NA	9	9-months
						DM: 27%			∙ in-stent restenosis: 13.0% (DM) vs 9.6% (non-DM), *p* = 0.12
									∙ 9-months late luminal loss: 0.40 mm vs 0.38 mm, *p* = 0.58
									12-months
									∙ TLR: 8.2% vs 4.9%, *p* = 0.02
									∙ ST: 0.8% vs 0.5%, *p* = 0.58
									∙ MACE: 15.9% vs 10.7%, *p* = 0.006
									∙ MI: 3.7% vs 3.6%, *p* = 0.90
									∙ Cardiac death: 0.7% vs 0.8%, *p * > 0.99
Sirolimus-eluting stents (SES)									
DIABETES [46]	RCT	2005	*De novo* lesions in native CAD	SES	BMS	80	80	4.5	∙ Mean late luminal loss: 0.06 mm ± 0.4 (SES) vs 0.47 mm ± 0.5 (BMS), *p * ≤ 0.001
						DM: 100%	DM: 100%		∙ In-stent late lumen loss: 0.09 mm ± 0.4 vs 0.67 mm ± 0.5; *p * ≤ 0.001
						IDDM: 32.5%	IDDM: 33.8%		∙ In-stent restenosis: 3.9% vs 31.7%; *p * ≤ 0.0001
									∙ MACE: 10% vs 36.3%, *p * ≤ 0.001
									∙ Need for revascularization: 6.3% vs 31.3%, *p * ≤ 0.001
									∙ Comparable results between IDDM vs non-IDDM
ISAR-DIABETES [62]	RCT	2005	*De novo* lesion	SES	PES	125	125	6	∙ LLL (in stent): 0.19 mm vs 0.45 mm, *p* = 0.001
						DM: 100%	DM: 100%		∙ LLL (in segment): 0.43 mm vs 0.67 mm, *p* = 0.002
									∙ Angiographic restenosis: 6.9% vs 16.5%, *p* = 0.03
DECODE [63]	RCT	2008	*De novo* lesions	SES (N = 54)	BMS (N = 29)	54	29	6	∙ LLL: 0.23 mm ± 0.54 (SES) vs 1.10 mm ± 0.59 (BMS), *p * < 0.001
						DM: 100%	DM: 100%		1-year
						IDDM: N = 10	IDDM: N = 6		∙ MACE (1-year): 14.8% vs 41.4%, *p* = 0.01
									∙ TVR (1-year): 3.7% vs 6.9%, *p* = 0.6
									∙ TVF (1-year): 14.8% vs 41.4%, *p* = 0.01
DESSERT [64]	RCT	2008	*De novo* lesions	SES (N = 75)	BMS (N = 75)	75	75	8	∙ LLL: 0.14 mm ± 0.33 (SES) vs 0.96 mm ± 0.61 (BMS), *p * < 0.001
						DM: 100%	DM: 100%		∙ In-segment binary restenosis: 3.6% vs 38.8, *p * < 0.001
						IDDM: 24%	IDDM: 27%		12-months
									∙ MACE: 22.1% vs 40%, *p* = 0.023
									∙ TLR: 5.9% vs 30%, *p * < 0.001
									∙ TVF: 14.7% vs 34.3%, *p* = 0.008
DiabeDES [65]	RCT	2009	*De novo* lesion	SES (N = 67)	PES (N = 63)	67	63	8	∙ LLL: 0.23 mm ± 0.54 (SES) vs 0.44 mm ± 0.52 mm (PES), *p* = 0.025
						DM: 100%	DM: 100%		∙ In-segment restenosis: 6.5% vs 11.8%, *p* = 0.25
						IDDM: 41%	IDDM: 38%		
DES-DIABETES [66]	RCT	2011	*De novo* lesions in native CAD	SES (N = 200)	PES (N = 200)	200	200	24	2-years
						DM: 100%	DM: 100%		∙ TLR: 3.5% (SES) vs 11.0% (PES), *p * < 0.01
						IDDM: 16%	IDDM: 16.5%		∙ TVR: 5.5% vs 12.0%, *p* = 0.01
									∙ MACE: 3.5% vs 12.5%, *p * < 0.01
									∙ Death: 0% vs 1.5%, *p* = 0.25
									∙ MI: 0.5% vs 1%, *p* = 0.99
									4-years
									∙ TLR: 7.0% vs 9.5%, *p* = 0.29
									∙ TVR: 8.0% vs 12.0%, *p* = 0.15
									∙ MACE: 11.0% vs 16.0%, *p* = 0.10
SCORPIUS [47]	RCT	2012	*De novo* lesions	SES (N = 95)	BMS (N = 95)	95	95	8	∙ Late luminal loss: 0.17 mm ± 0.45 (SES) vs 0.75 mm ± 0.59 (BMS), *p * ≤ 0.0001
						DM: 100%	DM: 100%		5-years
									∙ MACE: 36% vs 52%; HR: 0.6, 95% CI 0.4–0.9; *p* = 0.02
									∙ TLR: 13% vs 29%; HR: 0.4, 95% CI 0.2–0.7; *p* = 0.003
									∙ Death: 21% vs 21%
									∙ MI: 8% vs 9%
									∙ ST: 5% vs 6%
SIRTAX LATE [67]	RCT	2012	*De novo* lesion	SES	PES	503	509	60	Diabetics vs nondiabetics
						DM: 21.4%	DM: 18.2%		∙ MACE: 25.9% vs 19.2% (HR: 1.45, 95% CI 1.06–1.99, *p* = 0.02)
									∙ Cardiac death: 11.4% vs 4.3% (HR: 2.86, 95% CI 1.69–4.84, *p * < 0.0001)
									∙ TLR: 14.4% vs 14.1% (HR: 1.09, 95% CI 0.73–1.64, *p* = 0.67)
									No differences between IDDM (N = 64) vs non-IDDM (N = 137)
Zotarolimus-eluting stents (E-ZES)									
ENDEAVOR II [68]	RCT	2006	*De novo* lesion in native CAD	ZES	BMS	598	599	9	8-months
						NIDDM: 16.7%	NIDDM: 41.5%		TLR:
									∙ NIDDM: 6.3% vs 15.9%, *p* = 0.054
									∙ IDDM:11.5% vs 13.6%, *p* = 1.00
									In-stent binary restenosis:
									∙ Non diabetics: 7.8% vs 30.7% (HR: 0.25, 95% CI 0.15–0.42, *p * < 0.0001)
									∙ Non IDDM: 16.7% vs 41.5% (HR: 0.40, 95% CI 0.18–0.91, *p* = 0.002)
									∙ IDDM: 20% vs 47.4% (HR: 0.42, 95% CI 0.11–1.59, *p* = 0.25)
SCAAR registry [49, 50]	Prospective, observational registry	2009	All-PCI comers (N = 35,478)	NA	NA	SES: 2615	NA	48	∙ Restenosis
						PES Taxus Express: 2182			Diabetics vs nondiabetics (RR: 1.23, 95% CI 1.10–1.37)
						PES taxus Libertè: 2553			∙ Adjusted RR of restenosis in diabetics
						E-ZES: 881			E-ZES vs TAXUS Express: 2.08 (1.43–3.00)
									E-ZES vs TAXUS Libertè: 2.18 (1.55–3.07)
									E-ZES vs SES: 1.99 (1.43–2.77)
									Higher risk of restenosis with E-ZES both in the group of smaller stents (≤2.75 mm) and of larger diameter (>2.75 mm)
ZEST-Diabetes [69]	RCT	2010	*De novo* lesions	ZES	PES	883	PES: 884	24	MACEs
					SES	DM: 30%	DM: 27.7%		∙ Diabetics: 13.8% vs 7.7% vs 15.3%, *p* = 0.047
							SES: 878		∙ Nondiabetics: 10.3% vs 10.8% vs 15.3%, *p* = 0.011
							DM: 28.1%		Ischemia-driven TVR
									∙ Diabetics: 7.2% vs 1.7% vs 7.4%, *p* = 0.018
									∙ Nondiabetics: 10.3% vs 10.8% vs 15.3%, *p* = 0.011
NAPLES-DIABETES [70]	RCT	2011	*De novo* lesion	ZES	PES	75	PES: 75	36	MACE: 13.2% (SES) vs 17.5% (PES) vs 35.6% (ZES)
					SES	DM: 100%	DM: 100%		MACE-free survival:
							PES: 76		86.8% (SES) vs 82.5% (PES) vs 64.4% (ZES), *p* = 0.006
							DM: 100%		∙ No significant difference between SES vs PES (adjusted *p* = 1.0)
									Higher MACE in ZES vs SES (adjusted *p* = 0.012) and PES (adjusted *p* = 0.075).
SORT OUT III [51]	RCT	2012	*De novo* lesions	ZES	SES	1162	1170	18	Composite outcome
						DM: 14.5%	DM: 14.3%		∙ Diabetics: 18.3% vs 4.8% (HR: 4.05, 95% CI 1.86–8.82, *p* = 0.0004)
									∙ Nondiabetics: 8.3% vs 4.5% (HR: 1.87, 95% CI 1.30–2.69, *p* = 0.0008)
									TVR
									∙ Diabetics: 14.2% vs 3.0% (HR: 4.99, 95% CI 1.9–13.1, *p* = 0.0011)
									∙ Nondiabetics: 6.9% vs 3.4% (HR: 2.05, 95% CI 1.36–3.10, *p* = 0.0006)
									TLR
									∙ Diabetics: 12.4% vs 1.2% (HR: 11.0, 95% CI 2.59–47.1, *p* = 0.0020)
									∙ Nondiabetics: 5% vs 1.8% (HR: 2.85, 95% CI 1.67–4.8942, *p* = 0.0001)
ENDEAVOR IV [48]	RCT	2013	*De novo* lesion	ZES	PES	773	775	12	8 months in-stent late loss
						DM: 31%	DM: 30%		∙ Nondiabetics: 0.61 ± 0.44 vs 0.35 ± 0.39, *p * < 0.001
									∙ Diabetics: 0.81 ± 0.58 vs 0.56 ± 0.66, *p* = 0.073
									1-year TVF:
									∙ Nondiabetics: 7.4% vs 8.9%, *p* = 0.426
									∙ Diabetics: 8.6% vs 10.8%, *p* = 0.526
									MACE
									∙ Nondiabetics: 6.4% vs 6.4%, *p* = 1.00
									∙ Diabetics: 6.9% vs 7.2%, *p* = 1.000
									Independent of DM treatment
SECOND GENERATION									
Everolimus-eluting stents (EES)									
Stone *et al*. [23]	Pooled analysis of SPIRIT II, II, IV, COMPARE trials	2011	Native CAD	EES	PES	4811	1869	24	Diabetics (N = 1869) vs nondiabetics (N = 4811)
									∙ 2-year mortality: 1.9% vs 3.1%; *p* = 0.01
									∙ MI: 2.5% vs 5.8%; *p * < 0.0001
									∙ ST: 0.3% vs 2.4%; *p * < 0.0001
									∙ Ischemia-driven TLR 3.6% vs 6.9%; *p * < 0.0001
									No differences in diabetics
									Significant interactions between diabetic status and stent type for the 2-year end points of MI (*p* = 0.01), ST (*p* = 0.0006), and TLR (*p* = 0.02)
ESSENCE- DIABETES II [71]	RCT	2011	*De novo* lesions	EES	SES	149	151	8	In-segment late loss: 0.23 ± 0.27 vs 0.37 ± 0.52 mm; *p * < 0.001 for noninferiority
						DM: 100%	DM: 100%		in-stent restenosis: 0% vs 4.7%; *p* = 0.029
									in-segment restenosis: 0.9% vs 6.5%; *p* = 0.035
SPIRIT V [53]	RCT	2012	*De novo* lesions	EES	PES	218	106	9	∙ In-stent late loss: 0.19 mm vs 0.39 mm, *p* superiority = 0.0001
						DM: 100%	DM: 100%		∙ 1-year composite outcome (death, MI, and TVR): 16.3% vs 16.4%
									∙ 1-year ST: 0% vs 2%, *p* = 0.11
SORT OUT IV [72]	RCT	2014	*De novo* lesions	EES	SES	1390	1384	18	Composite endpoint
						DM: 14.0%	DM: 14.3%		∙ Diabetics: 10.3% vs 15.8% (HR: 0.63, 95% CI 0.36–1.11, *p* = 0.11)
									∙ Nondiabetics: 6.6% vs 6.3% (HR: 1.06, 95% CI 0.77–1.46, *p* = 0.71)
									TLR
									∙ Diabetics: 6.7% vs 10.7% (HR: 0.61, 95% CI 0.31–1.22, *p* = 0.16)
									∙ Nondiabetics: 4.5% vs 4.7% (HR: 0.96, 95% CI 0.66–1.39, *p* = 0.82)
TUXEDO INDIA [52]	RCT	2015	*De novo* lesions	PES	SES	914	916	12	∙ TVF: 5.6% (PES) vs 2.9% (SES) (RR: 1.89, 95% CI 1.20–2.99, *p* = 0.38 for noninferiority)
						DM: 100%	DM: 100%		Higher 1-year TVF in PES (*p* = 0.005), spontaneous MI (3.2% vs 1.2%, *p* = 0.004), ST (2.1% vs 0.4%, *p* = 0.002), TVR (3.4% vs 1.2%, *p* = 0.002), and TLR (3.4% vs 1.2%, *p* = 0.002).
Zotarolimus-eluting stents (R-ZES)									
TWENTE [73]	RCT	2012	All-comers	ZES	EES	697	694	12	TVF:
						DM: 22.7%	DM: 20.6%		∙ Diabetics: 7.7% (ZES) vs 13.9% (EES) (RR 1.81, 95% CI 0.91–3.60, *p* = 0.08)
									∙ Non-diabetics: 8.2% vs 6.5% (RR 0.80, 95% CI 0.52–1.22, *p* = 0.29)
RESOLUTE Global Clinical Program [74]	Pooled analysis from RESOLUTE FIM, All comers, International, US, and Japan	2017	All-comers	ZES	NA	5130	NA	12	∙ TVF: 12.1% vs 8.9%, *p* = 0.01
						DM: 29.9%			∙ TVR: 7.9% vs 5.3%, *p* = 0.01
									∙ Cardiac death or MI: 5.2% vs 4.1%, *p* = 0.20
									∙ MACEs: 11.3% vs 8.8%, *p* = 0.04
									∙ ST: 0.3% vs 0.4%, *p * > 0.99
BIONICS subanalysis [32]	RCT	2018	*De novo* lesions	RES	ZES	559	1360	12	∙ TLF: 7.8% vs 4.2%, *p* = 0.002
						DM: 100%	DM: 0%		∙ TVR: 4.5% vs 2.0%, *p* = 0.002
									∙ Restenosis: 15.2% vs 4.7%, *p* = 0.1
									∙ No differences in cardiac death or MI
BIORESORT [75]	RCT	2022	All-comers	ZES	SES	1173	SES: 1169	60	TVF: 21.1% (ZES) vs 19.8% (SES) vs 19.2% (EES)
					EES	DM: 17.9%	DM: 18.0%		∙ SES vs ZES (HR: 0.91, 95% CI 0.59–1.42, *p* = 0.69)
							EES: 1172		∙ EES vs ZES (HR: 0.90, 95% CI 0.58–1.40, *p* = 0.63)
							DM: 17.3%		
BIODEGRADABLE-POLYMER AND POLYMER-FREE									
De Waha *et al*. [76]	Pooled analysis from ISAR-TEST 3, 4, and LEADER trials	2013	*De novo* lesions	BP-DES	DP-SES	657	437	48	∙ Composite outcome: HR: 0.95, 95% CI = 0.74–1.21, *p* = 0.67
						DM: 100%	DM: 100%		∙ TLR: HR: 0.89, 95% CI = 0.65–1.22, *p* = 0.47
									∙ Definite or probable ST: HR: 0.15, 95% CI = 0.03–0.70, *p* = 0.02
COMPARE II [55]	RCT	2017	All-comers	BP-BES	DP-EES	1795	912	60	5 year-TVR:
						DM: 21.8%	DM: 21.6%		∙ Diabetics: 16% vs 11%, *p* = 0.09 (HR: 1.47, 95% CI 0.93–2.31)
									∙ Nondiabetics: 9% vs 8%, *p* = 0.62 (HR: 1.08, 95% CI 0.80–1.45)
									∙*p* for interaction = 0.29
									∙ IDDM: 23% vs 18%, *p* = 0.49 (HR: 1.29, 95% CI 0.67–2.27)
									∙ Non-IDDM: 10% vs 8%, *p* = 0.27 (HR: 1.16, 95% CI 0.89–1.51)
									∙*p* for interaction = 0.32
EVOLVE II-Diabetes Substudy [54, 77]	RCT	2017	*De novo* lesions	Synergy		263	203	12	∙ 1-year TLF: 7.5% vs 14.5% (*p * < 0.0002)
				BP-EES		DM: 100%	DM: 100%		∙ 2-years TLF: 11.2%, cardiac death 1.5%, MI 6.4%, TLR 6.8%, ST 1.1%
									∙ 5 years TLF: 17%
CENTURY II [57]	RCT	2018	*De novo* lesions	BP-SES	PP-EES	551	550	60	TVF: 13.6% (BP-SES) vs 11.8% (PP-EES) (RR 1.16, 95% CI 0.66–2.02, *p* = 0.86)
						DM: 31.9%	DM: 30.9%		
BIOFLOW-II [56]	RCT	2018	*De novo* lesions	BP-SES	DP-EES	298	154	60	∙ TLF: 15.9% vs 11.5% (HR: 1.43, 95% CI 0.51–4), *p* = 0.498
						DM: 29.5%	DM: 28.5%		∙ ST: 0% vs 6.9%, *p* = 0.039
						IDDM: N = 18	IDDM: N = 15		Cardiac death. 1.3% vs 6.9% (HR: 0.18, 95% CI 0.02–1.69, *p* = 0.089)
BIOSCIENCE subanalysis [56]	RCT	2019	*De novo* lesions	BP-SES	DP-EES	1063	1056	60	TLF
						DM: 24%	DM: 21.6%		Diabetics: 31% vs 25.8% (RR 1.23; 95% CI, 0.87–1.7, *p* = 0.24)
						IDDM: 8.4%	IDDM: 6.7%		Nondiabetics: 16.8% vs 16.8% (RR 0.98; 95% CI, 0.77–1.26, *p* = 0.90)
									No differences in cardiac death, target vessel-MI, clinically TLR, and definite ST in diabetics treated with BP‐SES or DP‐EES
Waksman *et al*. [78]	Pooled analysis from BIOFLOW II, IV, and V trials	2019	*De novo* lesions	BP-SES	DP-EES	494	263	12	∙ TLF:
						DM: 100%	DM: 100%		All diabetics: 6.3% (BP-SES) vs 8.7% (DP-SES) (HR: 0.82, 95% CI 0.047–1.43, *p* = 0.493)
						IDDM: 8.4%	IDDM: 10.5%		IDDM: 8.4% (BP-SES) vs 9.6% (DP-SES), *p* = 0.807
ISAR-TEST V Prespecified subgroup analysis [79]	RCT	2022	*De novo* lesions	PF-SES	DP-ZES	2002	1000	120	∙ MACE:
						DM: 28.7%	DM: 29.5%		Diabetics: 74.8% vs 79.6% (HR: 0.86, 95% CI 0.73–1.02; *p* = 0.08)
									Nondiabetics: 62.5% vs 62.2% (HR: 0.99, 95% CI 0.88–1.11; *p* = 0.88)
SORT OUT VII subanalysis [80]	RCT	2024	*De novo* lesions	O-SES	N-BES	1261	1264	60	∙ TLF
						DM: 18.7%	DM: 18.6%		Diabetics vs nondiabetics: 20.6% vs 11% (RR 1.85, 95% CI 1.42–2.40)
									Diabetics: 21.2% (O-SES) vs 20% (N-BES); (RR: 1.05, 95% CI 0.70–1.58, *p* = 0.81)
									∙ MACE
									Diabetics vs nondiabetics: 42% vs 31% (RR 1.43, 95% CI 1.19–1.71)
									No differences in cardiac death, MI, and TLR between O-SES and N-BES in diabetics
NEW GENERATION									
Cre8 EVO									
ASTUTE registry [81]	Prospective, observational registry	2016	All-comers	Cre8 EVO	NA	973	NA	12	∙ TLF
						DM: 41.8%			All cohort: 5.1%
						IDDM: 14.4%			Diabetics vs nondiabetics: 4.9 vs 5.3%, *p* = 0.788
									∙ TLR
									All cohort: 3%
									Diabetics vs nondiabetics: 3.7% vs 2.5%, *p* = 0.273
									No differences between IDDM vs non-IDDM
Cre8 SUGAR [58, 59]	RCT	2022	All-comers	Cre8 EVO	Resolute Onix DP-ZES (N = 589)	586	589	12	1-year
						DM: 100%	DM: 100%		∙ TLF: 7.2% vs 10.9% (HR: 0.65, 95% CI 0.44–0.9, *p* non inferiority < 0.001)
						IDDM: 31.2%	IDDM: 32.9%		∙ TVF: 7.5% vs 11.1% (HR: 0.67, 95% CI: 0.46–0.99; *p* = 0.042)
									∙ No differences in ST or cardiac death
									2-year
									∙ TLF: 10.4% vs 12.1% (HR: 0.84, 95% CI: 0.60–1.19; *p* superiority = 0.331)
									∙ TLR: 4.3% vs 4.5% (HR: 0.93, 95% CI: 0.54–1.60; *p* = 0.782)
									∙ Definite ST: 1% vs 1.2% (HR: 0.87, 95% CI: 0.29–2.58; *p* = 0.795)
									∙ MACE: 18.3% vs 20.8% (HR: 0.88, 95% CI: 0.68–1.16; *p* = 0.371)

Outcome data are reported as treatment vs control. Square brackets indicate 95% confidence intervals. Legend: BMS, Bare-Metal Stent; BP-EES, Biodegradable Polymer Everolimus-Eluting Stent; CAD, Coronary Artery Disease; DES, Drug-Eluting Stent; DM, Diabetes Mellitus; DP, Durable Polymer; E-ZES, Endeavor Zotarolimus-Eluting Stent; EES, Everolimus-Eluting Stent; IDDM, Insulin-Dependent Diabetes Mellitus; MACE, Major Adverse Cardiovascular Events; MI, Myocardial Infarction; N-BES, Nobori Biolimus-Eluting Stent; O-SES, Orsiro Sirolimus-Eluting Stent; PES, Paclitaxel-Eluting Stent; PF-SES, Polymer-Free Sirolimus-Eluting Stent; PP-EES, Permanent Polymer Everolimus-Eluting Stent; RCT, Randomized Controlled Trial; R-ZES, Resolute Zotarolimus-Eluting Stent; SES, Sirolimus-Eluting Stent; ST, Stent Thrombosis; TLR, Target Lesion Revascularization; TVF, Target Vessel Failure; TVR, Target Vessel Revascularization; ZES, Zotarolimus-Eluting Stent; TLF, target lesion failure.

### 4.2 First Generation DES: Paclitaxel, Sirolimus, and Zotarolimus Eluting Stents

While current-generation DES only releases limus derivatives, first-generation DES also included paclitaxel-eluting stent (PES) like TAXUS. In the TAXUS IV study, PES reduced MACE in patients with DM [45]. However, in a pooled analysis from the TAXUS Clinical Program involving 3513 patients, no differences were observed between PES and BMS in patients with DM regarding rates of death, myocardial infarction (MI), or stent thrombosis (ST) [82], despite lower rates of TLR over 4 years with DES—a consistent benefit observed in both insulin-treated (IDDM) and non–insulin-treated diabetic (NIDDM) patients. Similarly, trials evaluating a first-generation sirolimus-eluting stent (SES, CYPHER) yielded conflicting results [37, 46, 47, 83]. A meta-analysis of 3852 diabetic patients comparing SES, PES, and BMS confirmed that both DES types reduced mortality compared to BMS [84].

The interaction of drug choice and diabetes was explored in subsequent head-to-head comparisons between PES and SES. Paclitaxel works by disrupting microtubule function, and the influence of the metabolic alterations of T2DM appears limited [85]. At the same time, rapamycin analogs such as sirolimus inhibit cell cycle progression via glycosylation-dependent enzymes—mechanisms that may be less effective in the diabetic milieu [86]. Accordingly, it was noted that among patients receiving limus eluting stents, adverse events were lower in non-diabetics, intermediate in NIDDM, and higher in IDDM—a trend not observed in PES recipients [23]. However, angiographic data showed lower late lumen loss (LLL) and TLR with SES compared with PES [87, 88], suggesting a stronger antirestenotic effect.

Different from SES, the first zotarolimus-eluting stent (ZES, ENDEAVOR) initially showed noninferiority to PES [48], but subsequent data highlighted poorer outcomes among patients with DM, especially in IDDM [49, 50, 51, 89].

The excess risk of ST observed with first-generation DES, also evident in patients with DM, prompted the development of newer-generation devices [90].

### 4.3 Second Generation DES: Zotarolimus and Everolimus Eluting Stents

To address the limitations of ENDEAVOR, a second-generation ZES (RESOLUTE) was developed, featuring a prolonged drug-release profile, and it became the first DES specifically approved by the Food and Drug Administration (FDA) for use in patients with DM, although IDDM patients still showed an increased risk of target lesion failure (TLF) [91]. When comparing RESOLUTE to the second-generation XIENCE/PROMUS everolimus-eluting stent (EES) in 1855 patients with DM, 1-year event rates were low and comparable in the two groups (TLF: 3.5%) [92].

The thin, cobalt-chromium XIENCE/PROMUS EES has the broadest body of evidence from trials. Early studies demonstrated lower rates of restenosis, reduced neointimal hyperplasia, and lumen loss in patients with DM [93]. Clinical evidence showed that, in diabetic populations, EES was superior to first-generation SES and PES in both observational [49, 50] and randomized studies [52]. However, a subsequent pooled analysis found a higher rate of TLR with EES compared to PES in patients with IDDM [23]. A higher rate of TLR with EES versus PES was also found in the dedicated SPIRIT V Diabetic trial, in spite of overall similar clinical performance [53].

### 4.4 Biodegradable-Polymer and Polymer-Free Stents

To reduce the inflammatory response triggered by durable polymers (DP) of early DES, which contributes to delayed healing and late stent failure [94], biodegradable polymer coatings (BP-DES) were developed. However, clinical results were inconsistent: the SYNERGY stent, featuring a platinum-chromium strut and an abluminal bioabsorbable everolimus-eluting polymer, showed similar 5-year TLF rates as compared to PROMUS [54], but other platforms, such as ORSIRO (BP-SES) and XIENCE (BP-EES), were associated with higher TLR rates in diabetics (+106% and +55%, respectively) [95]. BP-DES and DP-DES showed similar outcomes in diabetic patients across several trials and meta-analyses [55, 56, 57, 96, 97]. Another large comparative analysis across second-generation DES platforms confirmed similar 3-year outcomes after risk adjustment [98]. The ultrathin Supraflex BP-SES is currently being compared to Xience EES in a diabetic population with MVD [99].

Given the suboptimal clinical outcomes of BP-DES, attention shifted to polymer-free DES, such as the Cre8 EVO SES. In this platform, the drug is stored in laser-drilled abluminal reservoirs, promoting targeted elution. In the Second-generation Drug-eluting Stents in Diabetes (SUGAR) trial, Cre8 EVO showed a lower 1-year target vessel failure (TVF) rate as compared to Resolute Onyx (DP-ZES) in diabetics [58]. However, this difference was no longer significant at the 2-year follow-up, and the study failed to demonstrate superiority [59].

## 5. Drug-Coated Balloons

### 5.1 Technology and Potential Benefits

Evidence is accumulating on drug-coated balloon (DCB) angioplasty for treatment of CAD [100]. In most cases, DCBs are semi-compliant balloons coated with a high-density antiproliferative drug that is released into the vessel wall during inflation without the need for a permanent metallic scaffold. Most DCBs are paclitaxel-coated (PCB), while a minority use sirolimus or its derivatives. Drug-release mechanisms change across different platforms, and drug pharmacokinetics is influenced by excipient, coating, and folding characteristics of the balloon. Hence, despite the absence of high-numerosity head-to-head comparisons, it is a common opinion that DCBs do not have a class effect and each device requires its own evidence of efficacy.

The absence of a permanent metallic frame and polymer may reduce the related vessel inflammation and neointimal proliferation, thereby potentially lowering restenosis rates. The risk of target lesion thrombosis is virtually erased, while avoiding vessel caging does not preclude positive vessel remodeling and late lumen enlargement. Moreover, DAPT after DCB-PCI might be shortened compared with DES, and the procedure is often simplified [100]. Although angiographically-evident dissections are common after DCB treatment, they do not correlate with adverse events [101]. Currently available evidence of DCB in T2DM is reported in Table 2 (Ref. [102, 103, 104, 105, 106, 107, 108, 109, 110, 111, 112, 113, 114, 115, 116, 117, 118, 119, 120, 121, 122, 123, 124, 125, 126, 127]), while **Supplementary Table 1** summarizes ongoing studies.

**Table 2.** **Main randomized and observational evidence on DCB in different clinical settings, according to diabetic status**.

Study	Study type	Year	Setting	Treatment	Control	Patients in the treatment arm (N) with % of DM and IDDM	Patients in the control arm (N) with % of DM and IDDM	Follow-up, months	Outcomes in diabetic patients (unless further specified)
In-stent restenosis									
PEPCAD-DES [104, 105]	RCT	2012	DES-ISR	SeQuent Please PCB	POBA	72	38	6	LLL:
						DM: 36.1%	DM: 34.2%		- DM: 0.51 ± 0.72 mm vs 1.45 ± 0.85 mm; *p * < 0.01
									- No DM: 0.39 ± 0.54 mm vs 0.91 ± 0.71 mm; *p * < 0.01
									TLR (36 months):
									- DM vs No DM in PCB cohort: 26.9% vs 15.2%; *p* = 0.23
									- DM vs No DM in POBA cohort: 38.5% vs 36%; *p* = 0.88
ISAR-DESIRE 3 [106, 107]	RCT	2013	DES-ISR	SeQuent Please PCB	Taxus PES or POBA	137	PES: 131	6–8	*p* for interaction between treatment and diabetic status: >0.34
						DM: 41%	DM: 47%		
						IDDM: 15%	IDDM: 21%		
							POBA: 134		
							DM: 37%		
							IDDM: 14%		
RIBS IV [108, 109]	RCT	2015/2018	DES-ISR	SeQuent Please PCB	Xience EES	154	155	6–9	MLD:
						DM: 49%	DM: 43%		- DM: nonsignificant difference (AMD: 0.08 mm; *p* = 0.49)
									- No DM: EES significantly better (AMD: 0.34 mm; *p* = 0.001)
									TLR (3 years):
									- DM: EES better (HR: 0.41)
									- No DM: EES better but nonsignificantly (HR: 0.46)
									*p* for interaction: 0.87
TIS [102]	RCT	2016	BMS-ISR	Sequent Please PCB	Promus Element EES	68	68	12	LLL:
						DM: 25.0%	DM: 24.5%		- DM: 0.12 ± 0.33 mm vs 0.48 ± 0.86 mm (*p* = 0.254)
BIOLUX [110]	RCT	2018	BMS- or DES-ISR	Pantera LUX PCB	Orsiro BP-SES	157	72	6	Consistent findings were observed in the diabetic and non-diabetic subgroups (details not reported)
						DM: 30.6%	DM: 33.3%		
DARE [111]	RCT	2018	BMS- or DES-ISR	Sequent Please PCB	Xience EES	137	141	6	MLD: no significant interaction with diabetic status
						DM: 42%	DM: 46%		
						IDDM: 15%	IDDM: 25%		
AGENT IDE [103]	RCT	2024	DES-ISR	Agent PCB	POBA	406	194	12	TLF:
						DM: 51%	DM: 50%		- DM: 21.6% in the PCB group vs 29.2% in the POBA group (HR: 0.71 [0.43–117], *p* = 0.18)
									- No DM: 15.2% in the PCB group vs 28.0% in the POBA group (HR: 0.50 [0.30–0.83], *p* = 0.006)
									*p* for interaction = 0.35
Small vessel disease									
BELLO [112, 113]*	RCT	2012	<2.8 mm	IN.PACT Falcon PCB	Taxus Libertè PES	90	92	6	LLL:
						DM: 43.4%	DM: 38.0%		- DM: 0.05 ± 0.41 mm vs 0.31 ± 0.51 mm; *p* = 0.033
						IDDM: 17.8%	IDDM: 9.8%		- No DM: 0.10 ± 0.36 mm vs 0.29 ± 0.40 mm; *p* superiority = 0.015
									*p* for interaction = 0.52
									TLR (12 months):
									- DM: 5.3% vs 13.9%, *p* = 0.205
									- No DM: 5.9% vs 5.4%, *p* = 0.906
BASKET-SMALL 2 [114, 115, 125]*	RCT	2018	<3 mm	Sequent Please PCB	Taxus Element PES and Xience EES	382	376	12	MACE:
						DM: 32%	DM: 35%		- DM: 10% vs 12%; HR: 0.83 [0.38–1.81]
						IDDM: 13%	IDDM: 13%		- No DM: 6% vs 5%; HR: 1.37 [0.64–2.90]
									*p* for interaction: 0.301
									TVR (3 years):
									- DM: 9.1% vs 15.0%; HR: 0.40 [0.17–0.94]
									- No DM: 8.75% vs 6.08%, HR: 1.64 [0.83–3.25]
									*p* for interaction = 0.011
PICCOLETTO II [116]	RCT	2020	>2 and <2.75 mm	Elutax SV/Emperor PCB	Xience EES	118	114	6	MACE:
						DM: 35.4%	DM: 38%		- DM: 9% vs 15%, HR: 1.17 [0.84–1.43]
						IDDM: 13.3%	IDDM: 17.8%		- No DM: 3% vs 3%, HR: 0.90 [0.65–1.21]
									*p* for interaction = 0.45
Long lesions									
Gitto *et al*. [117]	Observational	2023	LAD, 56 mm (mean length)	Several PCB and SCB	Several DES	139	139	24	DM was not significantly associated with TLF (HR: 1.99 [0.79–5.05]; *p* = 0.145)
						DM: 31.6%	DM: 23.0%		
Multivessel disease									
Her *et al*. [118]	Observational	2023	≥2 vessels treated	DCB-only or DCB + DES using Sequent Please PCB	Any DES	254	254	24	MACE:
						DM: 41%	DM: 45%		- DM: 2.9% vs 13.9%; HR: 0.19 [0.05–0.68]; *p* = 0.003
									- No DM: 4.7% vs 8.6%; HR: 0.52 [0.20–1.38]; *p* = 0.167
									TVR:
									- DM: 1.9% vs 7.0%; HR: 0.27 [0.05–1.34]; *p* = 0.077
									- No DM: 4.0% vs 5.8%; HR: 0.69 [0.23–2.07]; *p* = 0.492
*De novo*									
REC-CAGEFREE I [119]	RCT	2024	<60 mm, not requiring atherectomy nor Medina 1-1-1	Swide PCB	Firebird 2 SES	1133	1139	24	DOCE:
						DM: 24.9%	DM: 29.7%		- DM: 7.5% vs 3.9%, HR: 1.97 [0.99–3.94], *p* = 0.054
						IDDM: 5.9%	IDDM: 5.4%		- No DM: 6.0% vs 3.1%, HR: 1.94 [1.20–3.14], *p* = 0.0065
									*p* for interaction = 0.97
Ito *et al*. [120]*	Observational	2025	All comers	SeQuent Please PCB	NA	516	NA	30	MACE:
						DM: 51%			- DM vs No DM: 22.11% vs 11.9%. RR 1.86 [1.24–2.79], *p* = 0.002
						IDDM: 12%			TLR:
									- DM vs No DM: 10.6% vs 5.1% (RR 2.07 [1.10–3.91], *p* = 0.02)
Bifurcations									
DCB-BIF [121]	RCT	2025	SB lesion <10 mm and SB stenosis >70% after MV stenting	Any PCB	Any II gen DES	391	393	12	MACE:
						DM: 37.6%	DM: 35.6%		- DM: 7.8% vs 11.6%, HR: 0.66 (0.31–1.40)
						IDDM: 9.9%	IDDM: 7.6%		- No DM: 6.8% vs 13.0%, HR: 0.51 [0.29–0.92]
									*p* for interaction: 0.60
Acute coronary syndromes									
Merinopoulos *et al*. [122]	Observational	2023	STEMI without CA or CS	Any PCB	Any II gen DES	452	687	36	DM was a risk factor for mortality (HR: 2.13 [1.38–3.31], *p * < 0.001) regardless of treatment strategy at univariate analysis, but not at multivariable analysis
						DM: 14%	DM: 12%		
High bleeding risk									
DEBUT [126]	RCT	2019	One risk factor for bleeding	SeQuent Please PCB	Integrity BMS	102	106	9	MACE:
						DM: 26%	DM: 49%		- DM: 0% vs 19%, HR: 0.20 [0.05–0.87], *p* = 0.032
						IDDM: 9%	IDDM: 17%		- No DM: 1% vs 9%, HR: 0.68 [0.31–1.52], *p* = 0.68
REC-CAGEFREE II [127]	RCT	2024	Any ACS	PCB plus DAPT de-escalation	PCB plus 12-month DAPT	975	973	12	NACE:
						DM: 29.5%	DM: 31.6%		- DM: 12.2% vs 13.8%, HR: 0.89 [0.57–1.40], *p* = 0.63
						IDDM: 7%	IDDM: 8%		- No DM: 7.6% vs 6.3%, HR: 1.21 [0.81–1.82], *p* = 0.36
									*p* for interaction = 0.33
All comers									
NOBITRE registry [123]	Observational	2023	ISR or *de novo*	Any DCB (94% PCB)	Any DES	150	150	18	MACE: 21.6% vs 17.3%, aHR: 1.51 [0.46–4.93], *p* = 0.50
						DM: 100%	DM: 100%		TLF: 12.9% vs 9.4%, aHR: 5.6 [0.55–58], *p* = 0.15
Sirolimus vs paclitaxel-coated balloons for treatment of coronary artery disease	Observational	2025	ISR or *de novo*	Any SCB	Any PCB	990	330	12	TLF: no treatment effect between DM status and TLF (aHR: 1.00, 95% CI: 0.57–1.76)
						DM: 33.3%	DM: 30.3%		
EASTBOURNE Registry [124]*	Observational	2024	ISR or *de novo*	MagicTouch SCB	NA	2083	NA	12	MACE:
						DM: 41.5%			DM vs No DM: 12.2% vs 8.9%, HR: 1.26 [0.92–1.74]
						IDDM: 13.5%			TLR:
									DM vs No DM: 6.5% vs 4.7%, HR: 1.38 [0.91–2.08]

Only studies from 2012 onwards were included. Outcome data are reported as treatment vs control. Square brackets indicate 95% confidence intervals. Legend: AMD, adjusted mean difference; BMS-ISR, in-stent restenosis of bare-metal stent; DCB, drug-coated balloon; DES, drug-eluting stent; DES-ISR, in-stent restenosis of drug-eluting stent; DOCE, device-oriented composite outcome; DM, diabetes mellitus; HR, hazard ratio; IDDM, insulin-dependent diabetes mellitus; LLL, late lumen loss; MACE, major adverse cardiovascular events; MLD, minimal lumen diameter; NIDDM, non–insulin-dependent diabetes mellitus; PCB, paclitaxel-coated balloon; POBA, plain old balloon angioplasty; SB, side branch; SCB, sirolimus-coated balloon; SVD, small vessel disease; TLR, target lesion revascularization; TVF, target vessel failure; TVR, target vessel revascularization; NACE, Net Adverse Clinical Events. *Additional outcome data according to diabetic status are reported in the text.

### 5.2 Current Evidence and Outcomes

#### 5.2.1 In-Stent Restenosis

ISR was one of the first settings where DCB was implemented, in order to prevent stent-in-stent procedures and limit stent layers in the long term. The first report gave positive results following PCB versus POBA to treat BMS-ISR [128]. However, in spite of initial evidence showing comparable outcomes between DCB and DES for the treatment of ISR, subsequent trials and meta-analyses reported superiority of DES for the treatment of DES-ISR, making it the preferred option according to the 2024 ESC guidelines on chronic coronary syndromes [4, 129, 130]. However, the possible impact of DM on multiple stent layers is not taken into account in current guidelines.

Diabetic patients are fairly represented in major trials testing DCB in ISR (25–51%) [102, 103], but outcomes according to diabetic status were not frequently reported. The PEPCAD-DES (Treatment of DES-In-Stent Restenosis with SeQuent Please Paclitaxel Eluting PTCA Catheter) study found a lower LLL and larger minimal lumen diameter (MLD) at 6 months with PCB as compared to POBA in both diabetic and nondiabetic patients [104], with no differences in clinical outcomes at 3 years [105]. PCBs were noninferior to first-generation DES in the ISAR-DESIRE (Intracoronary Stenting and Angiographic Results – Drug Eluting Stent In-Stent Restenosis) 3 trial in terms of both angiographic and clinical outcomes up to 10 years, with no interaction with diabetic status [106, 107]. Conversely, in the RIBS (Drug-eluting balloons versus everolimus-eluting stents for patients with drug-eluting stent restenosis) IV trial, which used a second-generation DES as a comparison, the PCB arm had smaller MLD and higher rates of TLR [108, 109]. Recently, the AGENT IDE (A Clinical Trial to Assess the Agent Paclitaxel Coated PTCA Balloon Catheter for the Treatment of Subjects with In-Stent Restenosis) trial proved the superiority of a PCB over POBA in a contemporary (imaging used in >70% of cases) and complex (>40% of lesions were multilayer ISR) population [103]. The benefit was more evident in the nondiabetic than the diabetic subgroup, but without a statistically significant interaction.

Two studies included both BMS- and DES-ISR, reporting comparable angiographic and clinical outcomes with PCB as compared to a BP-SES and an EES in both diabetic and nondiabetic patients [110, 111]. Additional evidence is expected from ongoing RCTs (NCT05544864).

#### 5.2.2 Small-Vessel Disease 

Positive remodeling allowed by DCB and preserved vessel pulsatility have been advocated as their main benefit in the SVD setting.

The BELLO (Balloon Elution and Late Loss Optimization) trial compared a PCB with a PES in lesions with a diameter ≤2.8 mm, finding a similar MLD at 6 months [112]. In a post-hoc analysis reporting outcomes according to the presence of DM, DES conferred a larger MLD and greater acute gain at index procedure, but DCB yielded lower 6-month in-stent and in-segment LLL [113]. No significant differences in MLD, percentage of diameter stenosis, or net gain were found in patients with DM, as well as rates of restenosis and clinical outcomes. Overall, DCB were angiographically superior to DES in the diabetic cohort, but not in the nondiabetic one. Of note, the contemporary applicability of these results is limited by the use of a first-generation PES as the comparator arm.

The BASKET-SMALL (Basel Stent Kosten Effektivitäts Trial Drug Eluting Balloons vs Drug Eluting Stents in Small Vessel Interventions) 2 trial compared the Sequent Please PCB (Bbraun) with DES (Taxus PES first, then Xience EES when Taxus became unavailable) in vessels with a diameter <3 mm [114]. Noninferiority of PCB in terms of 1-year MACE was found. In patients with DM, although MACE did not differ between the two treatment strategies, target vessel revascularization (TVR) was significantly lower with DCB vs DES [115]. However, a sub-analysis including only EES (which represented 72% of the DES cohort) reported similar TVR rates among diabetic patients treated with DCB vs DES, although numerically higher in the DES cohort (12.5% vs 9.8%, HR: 1.85, 95% CI 0.74–4.66). A post-hoc analysis explored the impact of insulin treatment, which is a known risk factor for TLF after DES-PCI [131], and found IDDM patients (37.7%) had a higher risk of MACE as compared to NIDDM patients in both treatment groups [132]. TVR was numerically lower with DCB rather than DES in both IDDM (10.1% vs 15.7%, HR: 0.64, 95% CI 0.18–2.23) and NIDDM patients (8.4% vs 14.5%, HR: 0.30, 95% CI 0.09–1.03). A significant limitation of these trials is the lack of intravascular imaging guidance, which might result in an underestimation of actual vessel size, with an impact on DES-related adverse events.

The PICCOLETO (Drug Eluting Balloon Efficacy for Small Coronary Vessel Disease Treatment) II trial is a more contemporary study that tested a PCB vs Xience EES in SVD [116], showing similar 6-month LLL and 1-year MACE regardless of the presence of diabetes. A meta-analysis of these 3 RCTs and 3 Chinese studies showed that in diabetic patients with SVD, DCB was associated with lower MACE (HR: 0.60, 95% CI 0.40–0.91) and TLR (HR: 0.24, 95% CI 0.13–0.44) as compared to DES [133].

In a recent individual patient meta-analysis of the aforementioned studies, PCB reduced the risk of MACE at 3 years, while TLF rates were comparable. Diabetic status was an independent predictor of both outcomes, but did not significantly interact with the treatment effect [134].

#### 5.2.3 Long Lesions and MVD

**Fig. 2.**
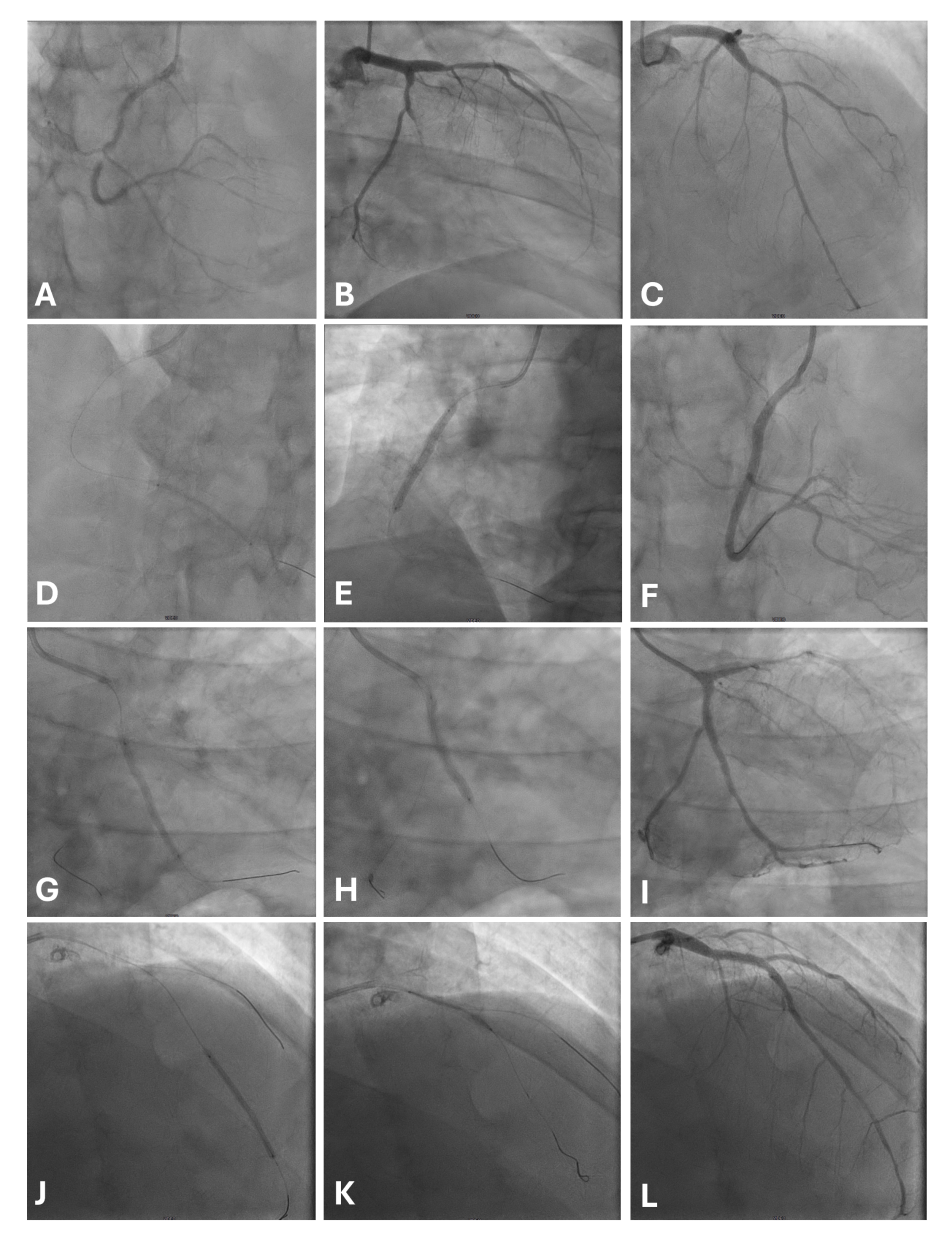
**A case of multivessel disease treated with a hybrid strategy (DES proximal, DCB distal)**. The patient was diabetic on metformin and was admitted for a non-ST elevation myocardial infarction with ongoing chest pain. Baseline angiogram shows a subocclusive stenosis of mid RCA, a critical stenosis of PDA (A), a calcific lesion of LCx extending toward OM, which has a thrombotic subocclusion (B), and a diffuse disease on LAD, with critical mid and distal lesions (C). After lesion preparation, RCA was treated distally with MagicTouch SCB (Concept Medical) 2.5 × 40 mm (D) and in the mid-segment with an Ultimaster Nagomi DES (Terumo) 3.5 × 38 mm (E), with a good final result (F). In the OM, flow was partially restored after wire crossing; after lesion preparation with an NC balloon, OM was treated with a MagicTouch SCB 3.0 × 40 mm (G) and LCx/proximal MO with a Ultimaster Nagomi DES 3.0 × 33 mm (H), with a final TIMI 3 flow (I). Distal LDA was treated with a MagicTouch SCB 2.50 × 40 mm (J), and an Ultimaster Nagomi DES 3.0 × 28 mm was implanted in the mid-segment (K). Final angiography showed a good result (L). Abbreviations: DCB, Drug-Coated Balloon; DES, Drug-Eluting Stent; LAD, Left Anterior Descending; LCx, Left Circumflex; NC, non-compliant; OM, Obtuse Marginal; PDA, Posterior Descending Artery; RCA, Right Coronary Artery; SCB, Sirolimus-Coated Balloon; TIMI, Thrombolysis In Myocardial Infarction (flow grade).

Diffuse and multivessel disease requires a considerable length of implanted stents, which is a well-known predictor of long-term thrombosis and restenosis [135]. In these settings, DCBs could be advantageous, allowing either to avoid DES implantation or to reduce stent length through a hybrid revascularization strategy (with proximal DES and distal DCB) (Fig. 2). Long LAD lesions have been explored only in a retrospective multicenter registry, which found a reduction in TLR and TLF with a DCB-based PCI, compared with a DES-only approach [117]. The presence of T2DM did not interact with the treatment effect. An RCT testing the hybrid strategy in diffuse disease is ongoing (NCT03589157). As for multivessel disease, a dedicated analysis found that patients with T2DM undergoing DCB-based PCI received a lower total stent length as compared to a DES-only approach (21.5 mm vs 64.9 mm for DES-only; *p* < 0.001) owing to a lower use of DES smaller than 2.5 mm (10.1% for DCB-based vs 42.6% for DES-only; *p* < 0.001) [118]. Among patients with DM, rates of MACE were lower in the DCB-based arm as compared to the DES-only arm, driven by fewer cardiac deaths and a numerically lower rate of TVR. In those without T2DM, no significant differences in MACE or its components were found, thus suggesting that the benefit of a DCB-based revascularization in multivessel CAD is more evident in patients with T2DM. As DCB use was not contemplated in the major trials confronting PCI with CABG in patients with DM and MVD, an updated trial testing a contemporary PCI approach is awaited.

#### 5.2.4 *De novo* Lesions in Large Vessels

In 2019, a meta-analysis of three studies found similar rates of MACE and TLR in 378 diabetic patients undergoing PCI with DCB or DES for *de novo* lesions [136]. However, only one of the three studies was an RCT, and a first-generation DES was the comparator arm. After encouraging observational evidence [137], the recent REC-CAGEFREE I RCT tested DCB versus DES in more than two thousand patients with short, noncomplex lesions [119]. DCB did not reach the prespecified noninferiority level, and this was consistent in both the T2DM (*p* = 0.054) and the non-T2DM subgroup. Notably, a significant treatment interaction emerged with vessel size: outcomes were similar in small vessels, but DCB use was associated with a 3-fold higher risk of TLF in large vessels compared to DES. Similar results were also found in bifurcation lesions and MVD. Altogether, this study suggests that DES may remain the standard-of-care for short, noncomplex lesions of large vessels, even in diabetic patients, since metal burden is limited.

A retrospective study on more than 500 Japanese patients treated with DCB found that T2DM almost doubled the risk of MACE, and IDDM almost tripled it [120]. Despite the limitations of the retrospective, nonrandomized design, this study has the merit of including more complex *de novo* lesions (including a third of DCB larger than 3 mm and almost half of lesions of B2/C type). Although results of this study might suggest that DCB is not effective in mitigating the residual risk of patients with DM, especially those with IDDM, this remains hypothesis-generating as lesion preparation was suboptimal as compared to recommended standards [100, 138]. The debate on DCB use on large *de novo* lesions (especially in complex settings [139]) is still open, and further research is ongoing—although not specifically focused on T2DM (NCT05550233, NCT05209412).

#### 5.2.5 Bifurcation

A DCB strategy for the side branch, or even the main branch when feasible, has the potential to simplify the procedure and mitigate the long-term risks associated with multiple overlapping metal layers. The DCB-BIF RCT found a lower incidence of MACE when a DCB was used to treat a side branch stenosis after main vessel stenting, as compared to a noncompliant balloon [121]. These results were consistent in both diabetic and nondiabetic patients.

#### 5.2.6 Acute Coronary Syndromes

Proper stent size could be underestimated during ACS, increasing the risk of stent malapposition or underexpansion [140]. The REVELATION (REVascularization with PaclitaxEL-Coated Balloon Angioplasty Versus Drug-Eluting Stenting in Acute Myocardial Infarction) trial found a DCB strategy noninferior to DES in terms of FFR value at 9 months but did not report outcomes according to diabetic status [141]. Another large prospective registry reported no differences in all-cause death, cardiac death, and unplanned TLR between STEMI patients treated with DCB-only or DES at 3 years [122]. Future evidence is awaited from RCT testing DCB and DES in the STEMI setting (NCT04072081,NCT06353594).

#### 5.2.7 All Comers

Recently, a PS-matched analysis confronting DCB- and DES-PCI in diabetic all comers found no difference in MACE but observed a significant reduction in overall mortality, which remains unexplained and may reflect bias from the observational design [123]. Moreover, a meta-analysis including 10 studies from different settings (mostly SVD and ISR) found that TLRs were significantly lower with DCB as compared to DES in diabetic patients (7.4% vs 10.9%; OR 0.66, 95% CI 0.44–0.99) [142].

#### 5.2.8 Different Drug, Different Effect?

The first RCT that compared sirolimus-coated balloons (SCB) versus PCB found comparable angiographic outcomes between the two balloons [143], but this result was not confirmed in the following TRANSFORM (Treatment of Small Coronary Vessels: MagicTouch Sirolimus Coated Balloon) I study, where PCB was superior in terms of late lumen loss at 6 months. Nonetheless, preliminary observational evidence suggests no difference in clinical outcomes between SCB and PCB at mid-term follow-up [144]. Of note, no RCT has reported T2DM-specific data. A recent analysis of the large, prospective EASTBOURNE (All-comer Sirolimus-coated balloon European) registry focused on outcomes of MagicTouch SCB in diabetic and nondiabetic patients [124]. Despite similar 1-year TLR and MACE rates, patients with DM had a higher incidence of spontaneous MI (3.4% vs 1.5% HR: 2.15, 95% CI 1.09–4.25). This analysis might suggest a positive effect of SCB on mitigating the added risk of T2DM on adverse events. More data on SCB in different settings will come from future RCTs (NCT04859985, NCT04893291).

## 6. Bioresorbable Vascular Scaffolds

Bioresorbable vascular scaffolds (BVS) were developed to reduce the chronic inflammation associated with the permanent presence of strut and polymer [145]. A pooled analysis of two RCTs found that patients with DM treated with everolimus-eluting BVS had similar 1-year outcomes compared to both non-diabetic BVS recipients and diabetic patients treated with EES in non-complex lesions [146]. Another pooled analysis reported a lower event rate with BVS as compared to a prespecified performance goal [147]. However, larger-scale RCTs stopped initial enthusiasm: although non-inferiority for the primary outcome was met in all studies, a high incidence of device thrombosis was reported (up to 3.5% at 2 years in one study) [148, 149, 150]. This raised safety concerns and led to device withdrawal in many countries. More recently, new scaffolds have been designed and are being tested in clinical practice. A magnesium BVS showed a good safety and efficacy profile in a large cohort of patients, with a 7.0% TLF rate at 2 years in patients with DM [151]. The same scaffold showed comparable results to DES in a small, nonrandomized cohort of patients with DM and ACS [152] (Table 3, Ref. [146, 147, 151, 153, 154, 155]). Nevertheless, in the absence of RCTs and evidence of benefit, these devices remain to be investigated.

**Table 3.** **Main evidence on BVS and hybrid devices**.

Study	Study type	Year	Setting	Treatment	Control	Patients in the treatment arm (N) with % of DM and IDDM	Patients in the control arm (N) with % of DM and IDDM	Follow-up, months	Outcomes in diabetic patients (unless further specified)
BVS									
Kereiakes *et al*. [147]	Pooled analysis from ABSORB II, III, JAPAN, and EXTEND registry	2017	*De novo* lesions	Absorb EES-BVS	NA	754	NA	12	∙ 1-year TLF: 8.3% vs 12.7%, *p* = 0.0001
						DM: 100%			
						IDDM 27.3%			
Muramatsu *et al*. [146]	Pooled analysis from ABSORB Cohort B, ABSORB EXTEND, and SPIRIT II-III-IV trials	2014	*De novo* lesions	ABSORB Cohort B (N = 101)	SPIRIT cohort	DM: N = 136	882	12	∙ Composite outcome:
				ABSORB EXTEND cohort (N = 450)	EES				Diabetics vs nondiabetics patients in the BVS group (3.7% vs 5.1%, *p* = 0.64).
									Diabetic BVS group vs diabetics in EES matched study group (3.9% vs 6.4%, *p* = 0.38).
									∙ ST:
									Diabetics vs nondiabetics patients in the BVS group (0.7% vs 0.7%)
									Diabetic BVS group vs diabetics in the EES matched study group (1% vs 1.7%)
BIOSOLVE-IV registry [151]	Prospective observational registry	2023	*De novo*, short lesions		NA	2066	NA	24	∙ TFL: 7.0% (vs 6.7% in non-DM, *p* = 0.770)
						DM: 444			∙ TV-MI: 1.8%
									∙ CD-TLR: 6.1%
Abluminus DES+									
En-ABLe-REGISTRY [153]	Prospective, observational registry	2018	All-comers	Abluminus DES+	NA	2500	NA	12	∙ 1-year MACE
						DM: 34.4%			Diabetics vs nondiabetics: 3.8% vs 2.2%, log rank = 0.051
						IDDM: 5.4%			IDDM vs non-IDDM: 5% vs 3.5%, log rank = 0.358
									∙ 2-year MACE
									Diabetics vs nondiabetics: 4.4% vs 2.4%, log rank = 0.025
									∙ 1-year ST
									Diabetics vs nondiabetics: 0.8% vs 0.4%, log rank = 0.363
ABILITY OCT REGISTRY [154]	RCT	2023	*De novo* lesions	Abluminus DES+	DP-EES	85	46	9–12	∙ Neointimal volume: 29.11 ± 18.90 mm^3^ vs 25.48 ± 17.04 mm^3^, *p* = 0.40
						DM: 100%	DM: 100%		∙ TLF: 21.2% vs 19.6%
									∙ TLR: 20% vs 17.4%
									∙ ST: 2.4% vs 0%
DEDICATE	Prospective, observational registry	On going		Abluminus DES+		3000		12	Ongoing
						DM: 100%			
ABILITY GLOBAL [155]	RCT	On going	*De novo* lesions	Abluminus DES+	Xience EES	1421	1447	12	Ongoing
						DM: 100%	DM: 100%		Preliminary results for non-inferiority:
									1-year TLR: 4.78% (DES+) vs 2.14% (EES), *p* = 0.409
									1-year TLF: 9.66% vs 6.25%, *p* = 0.658

Outcome data are reported as treatment vs control. Square brackets indicate 95% confidence intervals. Legend: BVS, bioresorbable vascular scaffold; DES+, sirolimus-coated hybrid drug-eluting stent and balloon system (Abluminus DES+); DP-EES, durable polymer everolimus-eluting stent; EES, everolimus-eluting stent; EES-BVS, everolimus-eluting bioresorbable vascular scaffold; IDDM, insulin-dependent diabetes mellitus; MACE, major adverse cardiovascular events; ST, stent thrombosis; TLF, target lesion failure; TLR, target lesion revascularization.

## 7. Next-Generation Scaffolds: Bioadaptive Stent Platforms

The DynamX® Sirolimus-Eluting Coronary Bioadaptor System (Elixir Medical Corporation) is a novel implantable device composed of 3 cobalt-chromium helical strands (71 mm) that are locked temporarily in delivery and acute expansion by a bioresorbable polymer. At 6 months, the polymer is reabsorbed, thus freeing the 3 struts, uncaging the vessel, and enabling late vessel expansion and remodeling. This should provide support to the vessel while enabling restoration of cyclic pulsatility. The reduced amount of metal should reduce adverse events after the first 6 months. Non-inferiority to conventional DES for TLF and TVF has been demonstrated in an all-comers population, with events plateauing after 6 months [156]. However, whether enabling adaptive remodeling and vasomotion might be of particular benefit in the diabetic population remains to be investigated [157].

## 8. Hybrid Devices 

Further innovation involves hybrid technologies combining DES and DCB features. The Abluminus DES+ (Concept Medical) is a novel, thin-strut BP stent with a sirolimus coating applied to both the stent and balloon. Upon deployment, the balloon extends 0.5 mm beyond the stent edges, enabling drug delivery to the lesion margins. This is a key target in patients with DM due to their increased risk of edge restenosis. The second innovative feature of this platform is the biphasic drug release: there is a peak phase over the first 3–4 days, followed by a stable, sustained release over 48 days.

In the first-in-human study, Abluminus DES+ demonstrated optimal 6-month LLL [158]. The en-ABL registry reported low 1-year MACE, slightly higher in patients with DM [153], while an optical coherence tomography found no difference in neointimal volume compared to DP-EES [154]. The ABILITY DIABETES GLOBAL trial is comparing Abluminus DES+ with Xience EES in 3050 patients from 21 countries. At the 1-year preliminary, unpublished results, the non-inferiority threshold for TLR was not met, partly due to unexpectedly low event rates in the control group [155] (Table 2).

## 9. Conclusions 

Despite substantial innovation and refinement in stent platforms, diabetic patients continue to experience a high rate of adverse events after PCI with DES implantation, underscoring a persistent residual risk related to both the baseline disease features and the permanent metallic scaffold apposition. Although still at an early stage of clinical use, emerging metal-limiting and metal-free alternatives may help improve outcomes after PCI in the complex lesion subsets that characterize this population.

## Abbreviations

ACS, acute coronary syndrome; CABG, coronary artery bypass grafting; CAD, coronary artery disease; BMS, bare metal stent; BVS, bioresorbable vascular scaffold; DCB, drug coated balloon; DES, drug eluting stent; DM, diabetes mellitus; MACE, major adverse cardiovascular events; MI, myocardial infarction; MVD, multivessel disease; ISR, intrastent restenosis; IVI, intravascular imaging; PCI, percutaneous coronary intervention; SMI, silent myocardial ischemia; STEMI, ST-elevation myocardial infarction; SVD, small vessel disease; T2DM, type 2 diabetes mellitus.

## Supplementary Material

Supplementary material associated with this article can be found, in the online version, at https://doi.org/10.31083/RCM44861.
