# Supplementary material for: Contemporary Percutaneous Coronary Intervention in Diabetic Patients
Source: Rev Cardiovasc Med. 2025 Dec 22;26(12):44861. doi: 10.31083/RCM44861 (PMC12781018; doi:10.31083/RCM44861)
Supplement: Supplementary file 1 [file 2153-8174-26-12-44861-s1.pdf]

SUPPLEMENTARY MATERIAL

Supplementary Table 1. Main ongoing RCT on DCB in T2DM in different clinical settings.

Legend: BMS-ISR: in-stent restenosis of bare-metal stent; DCB: drug-coated balloon; DES: drug-eluting stent; DES-ISR: in-stent restenosis of drug-eluting stent; DS%: diameter stenosis; EES: everolimus-eluting stent LLL: late lumen loss; MACE: major adverse cardiovascular events; MLA: minimal lumen area; MLD: minimal lumen diameter; NACE: net adverse cardiovascular events; PCB: paclitaxel-coated balloon; POBA: plain old balloon angioplasty; SB: side branch; SCB: sirolimus-coated balloon; SVD: small vessel disease; TL: target lesion; TLR: target lesion revascularization; TVF: target vessel failure; TVR: target vessel revascularization; US: united states; ZES: zotarolimus-eluting stent.

Study	Location	Setting	Diabetic patients	Treatment	Control	Sample size	Outcome	Follow-up, months	Status
**In-stent restenosis**									
**ISAR-DESIRE 5** (NCT05544864)	Germany, Spain	DES-ISR stratified into heterogeneous or homogeneous neointima assessed by OCT	Included	DCB	Xience DES	376	Primary: MACE<br>Secondary: TLF	24	Rectuiting
**MAGICAL ISR** (NCT05908331)	US	DES-ISR<br>Length: ≤36.0 mm	Included	MagicTouch SCB	POBA	492	Primary: TLF<br>Secondary: MACE	12	Rectuiting
**SELUTION4ISR** (NCT04280029)	US, Europe	BMS-ISR and DES-ISR	Included	Selution SCB	POBA or DES (ZES or EES)	418	Primary: TLF<br>Secondary: in-segment MLD	12	Completed
**SVD**									
**MAGICAL-SV** (NCT06271590)	US	Diameter: ≤2.75 mm	Included	MagicTouch SCB	DES (ZES or EES)	1065	Primary : TLF	12	Recruiting
**TRANSFORM II** (NCT04893291)	Europe, Bngladesh	Diameter: >2.0 mm and ≦3.5 mm<br>Length: 50 mm.	Included	MagicTouch SCB	DES (EES)	1820	Primary : TLF<br>Co-primary : NACE	12	Completed
**SELUTION 4 De novo Small Vessel IDE** (NCT05946629)	US	Diameter: 2.00-2.75 mm	Included	Selution SCB	Any DES	910	Primary: TLF	12	Recruiting
**AGENT Japan SV** (NCT04058990)	Japan	Diameter : ≥2.00 mm and <3.00 mm.<br>Length: ≤28 mm	Included	Agent PCB	Sequent Please PCB	180	Primary: TLF	6	Not recruiting
**A Safety and Efficacy Study of Dissolve in Treatment of Coronary Small**	China	Diameter : ≥2.25 mm and <2.75 mm.<br>Length: ≤26 mm	Included	Dissolve PCB	Resolute Integrity ZES	277	Primary : in-segment %DS<br>Secondary : device success	9	Completed

**Vessel Disease** (NCT03376646)									
**Long lesions**									
**Comparison of Safety and Efficacy of Coronary DCB Combined with Spot Stenting of DES Versus Second-generation DES for Treating Diffuse Coronary Artery Lesion** (NCT03589157)	China	Diameter : ≥2.5 mm and <4.0 mm. Length: >25 mm	Included	Any DCB	Any DES	140	Primary: LLL	9	Unknown
**Large vessels**									
**DCB-LVD** (NCT05550233)	China	Diameter : 3.0-4.0 mm	Included	Any DCB	Any DES	240	Primary: LLL Secondary: TLF	12	Unkwnon status
**CAGE-FREE III** (NCT05209412)	China	Diameter: 2.5-4.0 mm Length of a single lesion ≤ 35 mm; total treated lesion ≤ 60 mm;	Included	Lepu PCB	Resolute Integrity ZES	370	Primary: FFR Secondary: LLL	12	Not recruiting
**REVERSE** (NCT05846893)	Asia	Diameter : ≥3.0 mm	Included; pre-specified subgroup analysis according to DM status	Sequent Please PCB	Any DES	1436	Primary: NACE	12	Recruiting
**LARGE ONE** (NCT05961787)	China	Diameter : 3.0-4.0 mm. Length: ≤ 35 mm	Included	Sequent Please PCB	Firehawk DES	134	Primary: LLL Secondary: TLF	13	Recruiting
**Bifurcations**									
**Bingo Drug-eluting Balloon Versus a Drug-eluting Stent for Coronary Bifurcation Lesions** (NCT06441539)	China	Diameter MB: ≥2.5 mm and <4.0 mm. Length MB: <30 mm Diameter MB: ≥2.0 mm. Length MB: <20 mm	Included	Bingo PCB in both MB and SB	Xience EES in MB, POB or DES in SB	218	Primary: in-segment LLL	9	Not yet recruiting
**BJDCB-BIF** (NCT03223974)	China	Diameter MB: ≥2.5 mm and <3.5 mm. Length MB: <30 mm Diameter MB: 2.0-3.0 mm. Length MB: <22 mm	Included	PCB in both MB and SB	DES in MB	80	Primary: LLL and MLD	9	Completed

**Hybrid DEB** (NCT05731687)	Netherlands	MB nd SB diameter ≥ 2.5mm	Included	MagicTouch SCB in SB	Two-stent strategy	500	Primary: MACE	12	Recruiting
**ACS**									
**COPERNICAN** (NCT06353594)	Spain	STEMI (excluded CS)	Included	Any DCB on culprit lesion	Any DES	1272	Primary: TLF	12	Recruiting
**DCB versus DES in the Treatment of Coronary Artery Lesions in STEMI Patients in De Novo Coronary Lesions** (NCT04072081)	China	Diameter : 2.5-3.5 mm.<br>Length: ≤28 mm	Included	Any DCB	Any DES	4000	Primary: LLL	9	Unknown
**HBR**									
**PREPARE-NSE** (NCT03817801)	China	HBR	Included	NSE (Goodman) predilation + DCB (Sequent Please) treatment	NC balloon predilation + DCB (Sequent Please) treatment	60	Primary: MLA<br>Secondary: bailout DES rate	6	Completed
**De novo all-comers**									
**DCBinT2DM** (NCT07009938)	China	Diameter: 2.0-4.0 mm<br>Length: ≤40 mm	100%	Any DCB	Any DES	1000	Primary: TL DS%<br>Secondary: MACE	12	Not yet recruiting
**SELUTION DeNovo** (NCT04859985)	Europe	Diameter: 2.0-5.0 mm	Included	Selution SCB (+ provisional DES)	Systematic DES	3326	Primary : TVF	12 and 60	Completed
**FADDY** (NCT03452904)	China	Diameter : 2.5 mm to 3.5 mm<br>Length: <28 mm	Included	Any DCB	Any DES	80	Primary: FFR<br>Secondary: LLL	9	Unknown
**UNIQUE-DCB-I** (NCT04104854)	China	QFR: <0.8	Included	Any DCB	Any DES	220	Primary : LLL	12	Not yet recruiting
